# Impacts of paid sick leave policy adoption on heat-associated emergency department visit rates in two New Jersey municipalities: a quasi-experimental study

**DOI:** 10.3389/fpubh.2026.1923152

**Published:** 2026-10-22

**Authors:** Leah Schinasi, Augusta Williams, Joe Simeone, Claire Moore, Alina Schnake-Mahl

**Affiliations:** 1 Department of Environmental and Occupational Health, School of Public Health, Drexel University Dornsife, Philadelphia, PA, United States; 2 SUNY Upstate Medical University, Syracuse, NY, United States; 3 Drexel University Urban Health Collaborative, Philadelphia, PA, United States

**Keywords:** climate change, heat, occupational health & safety, policy, quasi-experimental research methodology

## Abstract

By supporting well-being, paid sick leave (PSL) policies may reduce heat vulnerability. A quasi-experimental difference-in-difference study of associations between early PSL policy adoption and heat-related morbidity outcomes in Jersey City and Newark, NJ, was conducted. Using data from the Healthcare Cost and Utilization Project State Emergency Department Database, a panel dataset of monthly counts of municipality-level all-cause emergency department (ED) visits among adults aged 18–64 from May to September 2011–2016 was constructed. The study linked these with monthly averages of daily minimum and maximum temperatures and was calculated using Oregon State University’s PRISM Group data. Multivariable fixed-effect Poisson models were used with panel Newey-West sandwich covariate estimators and finite-sample corrections to estimate associations between monthly average temperature and ED visit rates before and after PSL adoption. Models included the main temperature parameter; an interaction between temperature and an indicator for PSL adoption (pre-2014 = 0; 2014–2016 = 1); terms for monthly fine particulate matter (μg/m^3^) and precipitation (mm); and a population offset term. Rates of ED visits were 1.2% (95% CI: 0.995, 1.030) higher for every 1^0^ Celsius increase in monthly average maximum temperature, pre-policy enactment; associations were modestly attenuated post-policy enactment (rate ratio: 1.005, 95% CI: 0.993, 1.018). However, comparison municipalities, which did not adopt a PSL policy during the study period, experienced similar attenuation post-policy enactment, suggesting that associations might be explained by other factors, aside from the PSL policy. Additional research evaluating the impacts of PSL and other labor policies on heat vulnerability, with larger data sets and greater temporal granularity, is needed.

## Introduction

Unequivocally, ambient heat represents a critical environmental and occupational health hazard. Indeed, a large body of published research has documented links between high ambient temperatures and adverse morbidity and mortality outcomes in the general population and among vulnerable subpopulations, such as workers who labor in hazardous temperatures (1–4). The threats that heat presents to population well-being are not evenly distributed; heat is particularly dangerous for certain vulnerable subpopulations, defined and determined based on underlying biology, geography, exposure, and resources (5, 6).

Vulnerability, or the “…propensity to be adversely affected…” is a function of exposure intensity, biologic sensitivity, and adaptive capacity (7). Guided by evidence about the determinants of heat vulnerability, public health practitioners have developed messaging and interventions tailored to specific populations and geographical areas, with the goal of preventing heat-related health risks (8, 9). For example, health departments have developed or used heat vulnerability indices and maps to identify geographies where communities may have particular needs for the distribution of resources for heat adaptation (8, 10). Others have implemented heat warning systems, designed to reduce adverse health impacts in the event of extreme heat events by informing the need to undertake preventative measures (11, 12). Despite these efforts, hot temperatures continue to present health risks (13–15) and structural interventions are critically needed to reduce vulnerability and support health.

One such structural intervention is the enactment of workplace policies that allow heat-exposed workers to undertake critical preventative measures to reduce heat stress and associated adverse health outcomes (16). These preventative measures include having access to rest or hydration breaks, cooling stations, medical monitoring, altered work schedules, acclimatization periods, and heat safety trainings (17). As of this writing, in 2026, the U.S. does not have a federal occupational safety and health standard that specifically addresses workplace heat. Under the Biden-Harris administration, the U.S. Occupational Safety and Health Administration (OSHA) initiated the process to enact a federal standard (18), and a proposed rule was issued in August 2024. Public hearings were subsequently held in 2025 (19). However, a final rule has not been promulgated. In the absence of a federal policy, as of this writing in May 2026, eight states have enacted their own policies (20, 21). CA was the first state to enact such a policy—in 2005 (22). While few studies have analyzed employer compliance with and the health impacts of this policy, one investigation of CA agricultural workers found that, despite employer compliance with the CAL/OSHA heat safety law, some farm workers, such as those with chronic conditions, still experience overheating while working (23). These findings point to the need for broad social and labor policies or interventions that address additional determinants of heat vulnerability (24).

Social and labor policies have been shown to support population health and well-being and narrow health inequities (24). An exemplar policy is paid sick leave (PSL), which guarantees that workers receive pay when they take time off from work for short-term illness or injury or to engage in preventative care activities (e.g., visiting a primary care provider) for themselves or a family member (25). By supporting access to preventative health care and vaccine uptake (26–28), PSL may also support overall health and well-being and thus reduce sensitivity to heat (29, 30) (Figure). Indeed, having poor underlying health and/or chronic conditions, such as high body mass index, cardiovascular or respiratory disease, diabetes, or chronic kidney disease, can impact the body’s ability to dissipate heat via blood vessel dilation or sweating (29–31). In addition to reducing heat vulnerability by supporting overall health, PSL access may also prevent progression of heat illness, exacerbation of underlying chronic conditions, or on-the-job injuries resulting from heat-associated cognitive impairment—by allowing workers to stay home without fear of losing pay (24, 32, 33). Similar to workplace heat illness prevention policies, there is no federal PSL policy, though, as of this writing, eight state governments have enacted PSL policies since 2006 (BlueGreen (20, 21)).

In 2014, two municipalities in New Jersey (Jersey City, effective January 24; Newark City, effective June 21) adopted ordinances requiring employers to provide 1 h of PSL for every 30 h worked. The law applies to all workers employed for 80 or more hours in a calendar year (34, 35). These were the first PSL policies enacted in NJ municipalities. Jersey City’s policy allowed 40 h of accrual in a calendar year for all employees, regardless of firm size. Newark’s allows this same accrual for employers with 10 or more employees and 24 h of PSL for workers employed in firms with fewer than 10 employees.

This study leveraged this temporal (before versus after) and between-municipality variation in PSL policy adoption in NJ to bridge the gap between heat illness prevention efforts and social and labor policy enactment. Specifically, the study investigated whether the association between high ambient temperatures and ED visit rates in adults aged 18–64 was attenuated after these two municipalities adopted PSL policies. Then temporal trends in associations with demographically and climatologically similar NJ municipalities that did not enact a PSL policy during the same time-period were compared. The study hypothesized that PSL policy enactment would be associated with attenuated associations between heat and ED visits in Jersey City and Newark City and that any attenuation would be stronger in these two “treated municipalities,” as compared to similar NJ municipalities that did not adopt a PSL policy during the study period.

## Methods

### Identification of PSL effective dates

Two team members independently reviewed city websites to identify PSL effective dates and extracted the ordinances from the city website or Municode.com or ecode360.com if unavailable on the city website to determine when the municipality’s PSL policies went into effect. When discrepancies arose, the original reviewer returned to the legal text to reconcile them. Once returning to these, there were no remaining conflicts.

### Study setting and time-period

The study period for this analysis was determined by the dates on which the PSL ordinances became effective in Jersey City and Newark, NJ. Jersey City enacted its PSL ordinance on 25 September 2013, with an effective date of 24 January 2014. Newark enacted its ordinance on 28 January 2014, with an effective date of 21 June 2014 (34, 35).

This study included 3 years of data before and after 2014, the year PSL became effective in both municipalities, to allow for pre-treatment trends and allow for follow-up. The study period ended in 2016 to reduce concerns about selection bias and temporal confounding (36) and anticipatory effects associated with the enactment of a statewide PSL policy in NJ in 2018 (37). While several other NJ municipalities adopted PSL prior to the statewide policy, the treated areas were restricted to those that adopted the policy in 2014 to isolate impacts among early adopters and estimate the policy’s immediate impacts. Furthermore, the analyses were restricted to May–September to focus on morbidity outcomes in the warm season.

### Emergency department data

NJ ED visit data were used from the State Emergency Department Databases (SEDD), developed for the Healthcare Cost and Utilization Project (HCUP) (38), to construct a panel data set consisting of month-by-year-by-municipality-stratified counts of all-cause ED visits, 2011–2016. The NJ SEDD database captures discharge information on all ED visits to licensed, hospital-owned EDs that do not result in hospital admission. It excludes federally operated hospitals, such as those run by the U.S. Veterans Affairs. The HCUP SEDD data included the month and year of the ED visit, along with information on the person’s ZIP code and town of residence at the time of the ED visit.

The study population was restricted to ED visits among adults aged 18–64, with the goal of capturing the working and PSL-eligible population. All-cause ED visits were included as opposed to those with heat-specific codes because: (1) there were small numbers of ED visits coded as being attributable to heat exposure alone and (2) adverse heat-related outcomes can manifest in and be coded in a variety of ways, including cardiovascular, renal, respiratory, and injury mechanisms (13, 39, 40). Estimating associations with all-cause rather than cause-specific morbidity outcomes is consistent with the large body of existing epidemiologic evidence linking heat with all-cause outcomes (3, 41, 42). It is also well documented that estimating associations with outcomes identified using heat-specific codes under-represents the total burden of heat-related outcomes (43, 44).

### Weather and fine particulate matter (PM2.5) data

A 4-km resolution daily time-series data was used on ambient temperature (maximum and minimum, °C), dew point, and precipitation (mm), developed by Oregon State University’s PRISM Climate Group, to assign municipality-level monthly averages for each measure (45). The study also used the weathermetrics package (version 1.2.2) to calculate monthly averages of minimum and maximum daily relative humidity (46). Municipality-level estimates of fine particulate matter (PM_2.5_, μg/m^3^) were derived from the U.S. Environmental Protection Agency’s Downscaler model (47). The model uses air pollution levels measured at monitoring sites to calibrate estimates from the Community Multi-Scale Air Quality Model (CMAQ) onto a 12 km grid, yielding finer geographic resolution than monitoring sites alone provide. Daily tract-level PM_2.5_ estimates were linked to municipalities and aggregated into monthly averages.

### Study design

A difference-in-difference-in-temperature (DiDiT) design was used (48). This approach combines the difference-in-temperature (DiT) and the difference-in-difference (DiD) designs. Subsequently, a comparison group was added to conduct a DiDiDiT study. The DiT design exploits random, within-geography variation in year-to-year temperatures to estimate associations between monthly temperatures and ED visit rates. The underlying assumption of this approach is that year-to-year municipality fluctuations in monthly temperature are not related to other factors associated with ED visit rates. This approach has been used previously (48, 49). The DiD design is a robust, quasi-experimental, and well-accepted approach for evaluating population-level policies when randomized controlled trials are not feasible (36, 50).

In the DiT analyses, changes in rates of within-municipality ED visits associated with within-municipality fluctuations in monthly average temperatures (first difference) were estimated. Next, in the DiDiT formulation, post-policy adoption changes in the magnitude and direction of the slope representing the association between monthly temperature fluctuations and ED visit rates in Jersey City and Newark (second difference) were estimated. The study hypothesized that: (1) the DiT models would show that higher monthly ambient temperatures were associated with higher rates of ED visits, and (2) the DiDiT models would show that, post-policy adoption, the association between ambient temperatures and ED visit rates was attenuated. Finally, in the DiDiDiT formulation of this study, changes in the post- versus pre-policy adoption period associations between monthly temperatures and ED visit rates were compared in treated municipalities with these same associations in comparison with municipalities that did not adopt a PSL policy between 2011 and 2016 (third difference). The study hypothesized that any attenuated association between temperature and ED visit rates would be restricted to treated municipalities.

### Analysis

This study developed a series of fixed-effect models for the DiT, DiDiT, and DiDiDiT analyses. It identified evidence of overdispersion in the monthly ED visit count data and, as a result, first attempted to run negative binomial models. However, models did not converge. Thus, the study specified Poisson pseudo-maximum likelihood models (51) and used panel Newey-West heteroskedasticity- and autocorrelation-consistent sandwich estimators to estimate covariance matrices (52). In primary analyses, the study specified the Newey-West sandwich estimator using a bandwidth of two calendar months and indexed time using calendar year and month to ensure that September and the following May were treated as 8 months apart. Additionally, the study evaluated Newey-West bandwidths of 1, 3, 4, 8, and 12 calendar months in sensitivity analyses. All 95% confidence intervals were calculated using finite-sample adjustments, and *t* critical values were calculated using the small-sample correction in the fixest package. All models were run using version 0.12.1 of the fixest package (53).

#### DiT models

In the DiT models, municipality-specific, month-by-year averages of the minimum and maximum daily temperatures served as the independent variable. The study identified the optimal parameterization for temperature metrics by modeling them using linear, 2-degree-of-freedom (df) polynomial, or/and 2- or 3-df natural cubic spline terms and identifying the coding scheme that minimizes the BIC statistic. In both cases, a linear term provided the optimal model fit. Terms for monthly averages of precipitation and PM_2.5_ were included to adjust for potential confounding. Furthermore, the effect of additional adjustment for relative humidity on the magnitude of estimates of associations was evaluated; monthly average maximum daily relative humidity in models of maximum daily temperature and the monthly average minimum daily relative humidity in models of minimum daily temperature were included. Because the inclusion of relative humidity did not materially change the estimates of association between temperature and ED visit rates, the results from models without relative humidity, favoring a more parsimonious model (Appendix A), are presented. Models included fixed effects for month, year, and municipality and the natural logarithm of the municipality-level population aged 18–64 in the year 2013 (derived from 5-year American Community Survey estimates) as an offset. When exponentiated, the beta coefficient associated with the temperature term can be interpreted as estimating the relative change in ED visit rates per 1 °C increase in monthly temperature (either maximum or minimum, depending on the metric specified) within municipalities over time.

#### DiDiT models

Next, in the DiDiT models, an interaction term between the temperature parameter and a policy adoption indicator is included, coded 1 if the year was 2014 or later (the date after which the treated municipalities put the PSL policy into effect) and 0 otherwise. These models estimate changes in the association between temperature and ED visit rates, post-policy adoption. The interaction term quantifies the extent to which the beta coefficient estimating the association between temperature and ED visit rates was modified by policy enactment. Details on the DiT and DiDiT model specifications are given in Appendix B.

#### Comparison group

The study identified a group of NJ municipalities that did not adopt a PSL policy during the follow-up period to serve as a comparison group. To do so, the distributions of the following covariates in the baseline period (2011–2013) in the treated municipalities and in all other NJ municipalities that did not put PSL into effect before 2016 were calculated. The baseline covariates were 2013 population density, the percentage of the municipality living in poverty in 2013, and the average minimum and maximum temperatures during the warm season months in the baseline period. The study selected as a comparison group any municipality whose baseline characteristics fell within 10% (±) of the range of values for the treated municipalities. The final set of comparison municipalities was the following: City of Orange Township, Hackensack City, Harrison Town, Perth Amboy City, and Woodlynne Borough. Distributions of the baseline covariates for the treated and comparison municipalities are given in Appendix C. The study evaluated the sensitivity of the analyses to the 10% cut-point, which was chosen to balance the need for a sufficient number of untreated comparison areas with demographic and climatological similarities to treated municipalities. To do so, analyses using comparison municipalities whose baseline characteristics fell within 5, 20%, or 30% of the range of values for treated municipalities were repeated.

The study evaluated the assumption that pre-policy trends in the association between temperature and ED visit rates were similar in treated and comparison municipalities. To do so, analyses of the pre-policy adoption period were restricted and specified in models that included interaction terms between treated municipality status, calendar year, and the municipality-month average of daily minimum temperatures. The study evaluated associations with minimum rather than maximum temperatures because it was more predictive of ED visit rates, based on comparison of AIC statistics. This interaction term was small in magnitude and imprecise (*β* = −0.001350, Standard Error (SE) = 0.00374, *p* = 0.7), suggesting similar pre-policy trends in the temperature-ED visit rate association between treated and comparison municipalities. Event study models were estimated to further assess the pre-treatment trends by including interaction terms between event-time terms, monthly average minimum daily temperature, and an indicator term for treated municipality status, and plotting the corresponding coefficients. All the models were specified using a Poisson pseudo-maximum likelihood distribution and included fixed effects for municipality, month, and, in the model that did not include event-time terms, year. The models also included terms for month–year average precipitation, month–year average minimum daily temperature, and month–year average PM_2.5_ concentration. The event-time terms plot suggested little difference in the temperature-ED visit rate associations and no anticipation in the pre-policy period, as evidenced by estimates that were small in magnitude, imprecise, and included null. These estimates provide support for the parallel trends’ assumption (Appendix D).

#### DiDiDiT models

DiDiDiT models were run by including, in addition to the covariates included in the DiDiT models, interaction terms between a treated municipality indicator and the policy adoption indicator, a treated municipality indicator and the temperature metric, and a triple interaction term among all three variables. The coefficient associated with the triple interaction term estimates the extent to which the association between temperature and ED visit rates changed after policy enactment in treated municipalities relative to the corresponding change over the same period in comparison municipalities. Full details of the model are given in Appendix E.

#### Sensitivity analyses

In addition to the two sensitivity analyses mentioned above (testing of sensitivity to Newey-West bandwidth periods and of thresholds used to identify comparison municipalities), several other analyses were conducted to test the sensitivity of results to modeling decisions and analysis assumptions. To test for anticipation and lags in the impact of the policy, analyses were rerun, altering the timing of the policy enactment to 1 year prior to 2014 and 1 year after 2014 (2013 and 2015, respectively). In addition, analyses were run separately for each of the treated municipalities to evaluate whether one or the other was driving the results. Additionally, because Newark enacted its PSL policy in mid-June 2014, the analyses were rerun, specifying a policy effect start date of July 2014, as opposed than May 2014, as was done in the full analysis. The analyses for all ED visits across all age groups were rerun, removing the 18–64 age restriction. All analyses were conducted in R (version 4.5.2).

## Results

Total and median (interquartile range [IQR]) counts of ED visits in May–September were generally higher in treated than in comparison municipalities (Table 1), both before and after 2014. Across all municipalities, total and median (IQR) counts of all-cause ED visits were higher in the post- than pre-policy adoption period. For example, in Newark City, the median monthly total ED visits among those aged 18–64 was 68,642 (Interquartile Range [IQR]: 67,830, 71,470) in years 2011–2013 and 78,386 (IQR: 73,416, 80,273) in years 2014–2016. By contrast, in Hackensack City, a comparison municipality, the median monthly count of visits in years 2011–2013, was 7,315 (IQR: 7,007, 7,455) and 7,406 (IQR: 6986, 7,536) after PSL was adopted in Jersey City and Newark. Municipality-, month-, and year-specific ED visit counts are provided in Appendix F.

**Table 1:** Municipality-level counts of ED visits (all-cause) among adults aged 18–64 in the months of May–September in the years before and after PSL policies were adopted in Newark City and Jersey City (2011–2013 and 2014–2016, respectively).

Municipality	Pre-policy adoption		Post-policy adoption	
	Total	Median (IQR)	Total	Median (IQR)
Treated				
Newark City	1,040,844	68,642 (67,830, 71,470)	1,155,602	78,386 (73,416, 80,273)
Jersey City	532,210	33,411 (31,822, 39,186)	671,580	45,304 (43,145, 46,165)
Comparison				
City of Orange Township	68,355	4,564 (4,406, 4,714)	94,766	6,608 (5,408, 7,010)
Hackensack City	108,766	7,315 (7,007, 7,455)	109,228	7,406 (6,986, 7,536)
Harrison Town	23,660	1,540 (1,432, 1,712)	25,039	1,708 (1,610, 1,760)
Perth Amboy City	179,305	11,739 (11,700, 12,260)	195,678	13,090 (12,736, 13,296)
Woodlynne Borough	9,604	623 (592, 693)	9,289	609 (578, 665)

IQR, interquartile range.

In the pre-policy enactment period (2011–2013) (May–September) the median rate of ED visits was higher in treated than comparison municipalities (median: 2904.6/10,000 in treated versus 2,433.73/10,000 in comparison, Table 2). Temperatures were similar in both groups, with modestly higher values in treated municipalities. For example, the median of monthly average minimum daily temperatures, May–September, in years 2011–2013 was 18.2 °C in treated municipalities and 17.5 °C in comparison municipalities. Similarly, the population density and percentage living in poverty were higher in treated than comparison municipalities (median population density: 5498.12/km^2^ in treated, 4500.23/km^2^ in comparison; median percent living in poverty: 23.76 in treated, 22.02 in comparison).

**Table 2:** Baseline descriptive statistics (median, interquartile range) in treated and comparison municipalities, May–September, 2011–2013.

Characteristic	Median (interquartile range)		
	Overall	Untreated (*n* = 5)	Treated (*n* = 2)
ED visit rate per 10,000	2,433.73 [2,197.51, 3,446.51]	2,433.73 [2,377.65, 3,219.04]	2,904.55 [2,460.96, 3,348.15]
Population density per square km	4,500.23 [4,319.44, 5,249.00]	4,500.23 [4,212.43, 5,229.99]	5,498.12 [4,962.28, 6,033.96]
Maximum temperature (°C)	26.76 [26.46, 26.99]	26.90 [26.68, 27.08]	26.42 [26.25, 26.59]
Minimum temperature (°C)	17.74 [17.36, 18.15]	17.54 [17.18, 17.77]	18.18 [17.96, 18.40]
Precipitation (mm)	4.48 [4.43, 4.53]	4.52 [4.48, 4.54]	4.43 [4.43, 4.44]
PM2.5 (μg/m^3^)	10.55 [10.43, 10.71]	10.48 [10.39, 10.55]	10.84 [10.75, 10.94]
Percent living in poverty	22.02 [18.02, 26.55]	22.02 [17.65, 24.38]	23.76 [21.07, 26.44]

### DiT analysis results

Table 3 shows results from the DiT analyses estimating associations of monthly average minimum or maximum daily temperatures with rates of ED visits in adults aged 18–64 in Jersey City and Newark, NJ. On average, between May and September in years 2011–2016, each 1-degree Celsius higher monthly average of minimum and maximum daily temperatures was associated with approximately 1% higher rates of ED visits (95% CI for minimum daily temperatures: 0.995, 1.030, Table 3).

**Table 3:** Rate ratio (RR) and 95% confidence interval estimates (LL, Lower limit; UL, Upper limit) of the association between monthly averages of minimum and maximum daily temperatures and rates of ED visits among those aged 18–64 May–September, 2011–2016, in Jersey City and Newark City, NJ.^1^

Parameter	RR	LL	UL
Average minimum daily temperature	1.013	0.995	1.030
Average maximum daily temperature	1.006	0.994	1.019

^1^Results are derived from year, municipality, and month-fixed effect, Poisson-distributed models of the association between the municipality-, month-, and year-specific ED visit counts in those aged 18–64 and the municipality, month, and of daily minimum and maximum temperature, adjusted for average monthly precipitation, and month–year average PM2.5 concentration. Models included the natural logarithm of the aged 18–64 2013 population in the municipality as an offset term. All 95% confidence intervals were calculated using the calendar-gap-aware Newey-West inference, the fixest 0.12.1 small-sample correction, and the model-specific Student’s *t* critical value.

### DiDiT analysis results

Table 4 shows results from the DiDiT analyses estimating modification of the association between monthly averages of daily temperature and ED visit rates by PSL policy adoption. Associations and changes were modest. After the PSL policy was implemented in 2014, the association between maximum temperature and ED visit rates was modestly attenuated toward null. For example, the rates of ED visits were 1.2% (95% CI: 0.995, 1.030) higher for every 1 °C increase in the monthly average of maximum daily temperatures before the policy was put into effect (years 2011–2013) and 0.5% higher (95% CI: 0.993, 1.018) after its effect date (years 2014–2016). This represents a 0.7% decrease in the rate ratio representing the association between monthly averages of maximum daily temperature and ED visit rates (95% CI: 0.980, 1.006). The association between minimum temperature and ED visit rates remained unchanged, post- versus pre-policy effect date.

**Table 4:** Rate ratio (RR) and 95% CI estimates (LL, Lower limit; UL, Upper limit) of associations between monthly averages of minimum and maximum daily temperatures and rates of ED visits among those aged 18–64 in NJ, May–September 2011–2016, before and after 2014, when PSL policies were adopted in Jersey City and Newark City, NJ.^1^

Parameter	RR	LL	UL
Minimum temperature			
Pre-enactment DIT	1.013	0.992	1.033
Post-enactment DIT	1.013	0.995	1.031
Difference in post- versus pre-enactment DIT	1.000	0.987	1.013
Maximum temperature			
Pre-enactment DIT	1.012	0.995	1.030
Post-enactment DIT	1.005	0.993	1.018
Difference in post- versus pre-enactment DIT	0.993	0.980	1.006

^1^Results are derived from DiDiT analyses estimating the modification of the association between municipality-specific monthly averages of daily temperature and monthly emergency department (ED) visits in adults aged 18–64 in Jersey City and Newark City, NJ. Models were specified as Poisson distributed and adjusted for municipality-month- and year-specific averages of daily minimum and maximum temperature, an interaction term between the temperature metric and an indicator for PSL adoption (years 2011–2013 = 0, 2014–2016 = 1), average monthly precipitation, month–year average PM2.5 concentration, and fixed effects for year, municipality, and month. Models include the natural logarithm of the age 18–64 2013 population in the municipality as an offset term. All 95% confidence intervals were calculated using the calendar-gap-aware Newey-West inference, the fixest 0.12.1 small-sample correction, and the model-specific Student’s *t* critical value.

### DiDiDiT analysis results

Results from the DiDiDiT analysis comparing post- versus pre-adoption changes in the association between monthly average minimum and maximum daily temperatures and ED visit rates are given in Table 5. Associations and post- versus pre-policy period changes were very small in magnitude. The temperature–ED visit association in comparison municipalities following policy enactment was modestly attenuated (post- versus pre-policy difference: 0.989, 95% CI: 0.978, 1.000), whereas in the treated municipalities, the association was not different (difference in post- versus pre-enactment difference: 1.000, 95% CI: 0.989, 1.011). As a result, the DiDiDiT estimator for associations with monthly averages of minimum temperature was 1.011 (95% CI: 0.998, 1.024), reflecting extremely modest differences in the DiDiT estimators in treated and comparison municipalities.

**Table 5:** Rate ratio (RR) and 95% CI estimates (LL: lower limit, UL: upper limit) of associations between monthly averages of minimum and maximum daily temperatures and rates of ED visits among those ages 18 to 64 in NJ, May-September, 2011-2016, before and after 2014, when PSL policies were adopted in Jersey City and Newark City, NJ^1^, in treated versus comparison municipalities.

Parameter	Rate ratio	95% CI	
Minimum temperature			
Pre-enactment DiT, comparison	1.016	1.000	1.032
Pre-enactment DiT, treated	1.010	0.995	1.026
Post-enactment DiT, comparison	1.005	0.989	1.021
Post-enactment DiT, treated	1.010	0.998	1.023
Difference in post- versus pre-enactment slopes, comparison	0.989	0.978	1.000
Difference in post- versus pre-enactment slopes, treated	1.000	0.989	1.011
DiDiDiT estimate	1.011	0.998	1.024
Maximum temperature			
Pre-enactment Dit, comparison	1.015	1.001	1.029
Pre-enactment Dit, treated	1.012	0.999	1.026
Post-enactment DiT, comparison	1.004	0.991	1.018
Post-enactment DiT, treated	1.005	0.996	1.015
Difference in post versus pre-enactment slopes, comparison	0.990	0.978	1.002
Difference in post versus pre-enactment slopes, treated	0.993	0.982	1.004
DiDiDiT estimate	1.003	0.990	1.016

^1^Results are derived from DiDiDiT analyses estimating a modification of the association between municipality-specific monthly averages of daily temperature and monthly ED visits in adults aged 18–64 in Jersey City and Newark City, NJ. Models were specified as Poisson distributed and included covariates for municipality, month, and year-specific averages of daily minimum or maximum temperature; an interaction term between the temperature metric and an indicator for PSL Policy enactment (years 2011–2013 = 0, 2014–2016 = 1); an interaction term between the PSL indicator and treatment status (1 = treated, 0 = comparison); a three-way interaction term between the temperature metric, the PSL indicator, and treatment status; average monthly precipitation; and month-year average PM2.5 concentration; and fixed effects for year, municipality, and month. Models included the natural logarithm of the 2013 population aged 18–64 in the municipality as an offset term. All 95% confidence intervals were calculated using calendar-gap-aware Newey-West inference, the fixest 0.12.1 small-sample correction, and the model-specific Student’s *t* critical value.

### Sensitivity analyses

The DIT estimates were modestly closer to null when it was falsely specified that the PSL policy was enacted in either 2013 or 2015. When the policy effect start date was set to be July 2014, rather than May 2014, the analysis results remained unchanged. DiDiT analysis results were similar for each municipality, separately, and similar to the pooled DiDiT results. Results were also similar when associations with all ED visits were evaluated (i.e., including those that occurred in people who were either younger than 18 or older than 64) (Appendix G). Results were also similar when analyses were conducted using as comparison municipalities those for whom baseline covariate distributions fell within ±5, 20, and 30% of the range of values for treated municipalities, rather than 10% (Appendix H). Finally, when Newey-West bandwidths of 1, 3, 4, 8, and 12 calendar months were evaluated, instead of 2 calendar months, in sensitivity analyses, a little change in estimates was noticed (Appendix I).

## Discussion

In the face of hazardous, hot temperatures, structural interventions in the form of policies and programs are needed to support health and reduce population vulnerability. In this analysis, we found that higher monthly averages of daily temperatures were modestly associated with higher rates of ED visits among working-age adults in two municipalities in NJ. There was evidence of modest attenuation of this association once a PSL policy went into effect. However, we observed a similar (or, if anything, a modestly larger) attenuation in the association between high monthly ambient temperatures and ED visit rates in five municipalities that did not enact a PSL policy during the follow-up period, suggesting that any observed attenuation could be explained by other temporal trends.

Our findings suggesting that incrementally higher temperatures were associated with higher ED visit rates are consistent with a large body of evidence showing links between hotter temperatures and acute morbidity outcomes, including asthma exacerbation, cardiovascular events, dehydration, and injury (2, 13, 54, 55). For example, one study found that an additional day of 100 °F heat index within a week was associated with higher use of the ED (56). There are a variety of mechanisms by which heat may lead to morbidity. Briefly, when high ambient temperatures cause the body to overheat, the human body responds by dissipating heat through a variety of physiological mechanisms (57). These include sweating and blood vessel dilation, mediated by cardiovascular and autonomic responses (58). When the body is unable to sufficiently dissipate heat, body temperatures continue to rise. This can lead to illness or death (30). Those with underlying chronic conditions, such as cardiovascular, respiratory, and renal disease, are particularly sensitive to heat and may also have impaired ability to release heat, making them susceptible to adverse morbidity outcomes (30).

PSL access is associated with increased uptake of health-promoting behaviors, such as vaccination or visiting a healthcare professional when sick (59). We hypothesized that, by supporting better preventative care, improving underlying health, and, thus, reduced sensitivity to heat, PSL would be associated with attenuates associations between heat and ED visit rates (24). Another mechanism by which PSL access might reduce heat vulnerability is by allowing people with underlying conditions to call out sick during high heat days, thus limiting heat exposure (26).

To our knowledge, no other study has evaluated this specific hypothesis. Past research has shown that access to healthcare is associated with attenuated heat-associated mortality outcomes. For example, across US counties, improved community health care center access in the 1960s and 1970s was associated with a 14.2% relative decrease in the association between heat and higher rates of mortality (60). In Korea, greater access to healthcare resources (pre-hospital EMS, hospital resources, and timely access) was associated with attenuating heatwave-related mortality rates (61).

We acknowledge several limitations, some of which may explain our modest or null findings. We initially intended to study occupational outcomes, defined as ED visits coded as paid through workers’ compensation. However, ED visit counts paid using workers’ compensation were underpowered to support this analysis. We, therefore, defined our outcome, in the main analysis, as ED visits in the working-age population (adults ages 18–64). This assumes that all people ages 18 to 64 were working and that the population we captured was exposed to the ambient temperatures captured in this analysis. It is unlikely that, in all cases, these assumptions were met. In general, the data limitation points to a critical gap in occupational health research, which is access to comprehensive surveillance data for workers, and particularly the most socially vulnerable workers, who may also be the most heat vulnerable and exposed (24, 62–64). Even with our more expansive definition of outcomes, we were likely underpowered to detect true associations with ambient heat. Indeed, we limited our analysis to two treated municipalities and our follow-up period to 2011–2016. While these restrictions may reduce concerns about biases due to anticipatory effects and selection, they also led to small numbers. Thus, we stress the importance of replicating this study with larger data sets and improved outcome ascertainment. Our approach also assumes that all those aged 18 to 64 living in the ‘treated municipalities’ had access to PSL once the policy took effect. However, this assumption may not always hold. For example, construction workers covered by collective bargaining agreements, people working as per diem healthcare workers, and those who were independent contractors were excluded from coverage in Jersey City (35). Those employed by any school district or board of education or who were members of a construction union covered by a collective bargaining agreement were excluded from coverage by Newark’s law (34). Furthermore, many workers may already have had PSL access through employers or collective bargaining agreements prior to the policy effective dates. The overlap in PSL access, before and after the policies took effect would attenuate of the estimates of association toward the null. Furthermore, from the ED data, we had information only on the municipality of residence. Because the PSL policies applied to those employed in the city regardless of residence, policy exposure was potentially misclassified for individuals who lived but did not work in a treated municipality and for those who worked in a treated municipality but lived elsewhere. Similarly, temperature and air pollution exposures reflected residential, as opposed to occupational environments. We assume that any such misclassification would be non-differential with respect to the outcome and expect it would result in attenuation of the estimates of the modifying effect of PSL adoption on the association between heat and ED visit rates toward the null. Another concern is that, because of limited information in the ED data, we were unable to stratify or restrict analyses to individuals working in heat-exposed industries. However, we hypothesize that PSL may support heat resilience at a population level. Even those who are not directly exposed to heat at work may be highly vulnerable to heat, for example, in the home environment. Being able to take paid time off work to support health, well-being, and recovery might reduce heat vulnerability for everyone, even if they are not exposed to heat at work.

We were also limited by the temporal resolution of the outcome data. AHRQ releases information only on the month and year of the ED visit. Thus, we were unable to investigate acute associations between daily ambient temperature and daily ED visit rates. The relatively coarse temporal resolution of the ED visit and temperature data may have obscured important relationships between acute spikes in heat and morbidity outcomes; past studies have shown relatively short lag periods between temperature spikes and mortality outcomes (2, 65, 66). Furthermore, the SED dataset only includes ED visits that did not result in hospital admission. As a result, our analysis excludes more severe encounters that resulted in hospitalization. In addition, PSL access may have increased ED utilization after policy adoption by reducing barriers to seeking medical care. If this is the case, then our findings may reflect improved access to treatment for heat-associated morbidity.

Despite these limitations, this analysis has important strengths. We employed a robust quasi-experimental study design. We conducted analyses that included a comparison group and also conducted a number of sensitivity analyses to test the robustness of the results. We estimated associations with several temperature metrics and adjusted for important time-varying covariates. We included year fixed effects in our models to account for statewide temporal changes, such as the adoption of statewide or federal policies or programs, that affected all municipalities similarly during the follow-up period. More generally, this analysis is valuable because it highlights the potential importance of broad social and labor policies in reducing heat vulnerability. More research is needed to investigate the impacts of paid sick leave and other social and labor policies on heat vulnerability among workers and the general population. Future studies should explore these associations using larger data sets with more granular temporal information on acute healthcare encounters.

## Conclusion

Hot temperatures are a pressing population health threat. A healthy and supported population, with access to critical social determinants of health, will be well-equipped to survive the figurative and literal storm of extreme weather exposure. In addition to policies that intervene to reduce heat exposure and support adaptive capacity, such as workplace heat safety policies, it is important to consider structural interventions that support general well-being and health and, thus, may reduce heat sensitivity (24). In this analysis, the hypothesis that PSL policies may attenuate adverse heat-associated morbidity outcomes in working-age adults was investigated. While this study did not produce convincing evidence to support this hypothesis, future research on links between PSL, other labor policies, and heat vulnerability in larger data sets and with improved outcome ascertainment is critically needed to support well-being in the face of a warming world.

## Data Availability

Publicly available datasets were analyzed in this study. This data can be found here: https://hcup-us.ahrq.gov/seddoverview.jsp.
