# Supplementary material for: Impacts of paid sick leave policy adoption on heat-associated emergency department visit rates in two New Jersey municipalities: a quasi-experimental study
Source: Front Public Health. 2026 Oct 22;14:1923152. doi: 10.3389/fpubh.2026.1923152 (PMC13736741; doi:10.3389/fpubh.2026.1923152)
Supplement: Supplementary file 1 [file Data_Sheet_1.PDF]

Appendix A. Rate ratio (RR) and 95% CI estimates of association between monthly averages of minimum and maximum temperatures and rates of ED visits among those ages 18 to 64 in NJ, May-September, 2011-2016, before and after policy enactment, in NJ City and Newark City, NJ. In these sensitivity analyses, monthly average of minimum daily relative humidity, in models with minimum daily temperature as the estimator, and maximum daily relative humidity, in models with maximum daily temperature as the estimator, were included as an additional covariate.

Sensitivity analysis	Sample	Temperature metric	Estimator	RR	LL	UL
Additional relative-humidity adjustment	2 treated Municipalities	Minimum temperature	Temperature slope	1.010	0.993	1.027
		Maximum temperature	Temperature slope	1.007	0.995	1.020

RR = rate ratio; LL = lower limit; UL = upper limit. All 95% confidence intervals use calendar-gap-aware Newey-West inference, the fixest 0.12.1 small-sample correction, and the model-specific Student's t critical value.

Appendix B. Details on DiD and DiDiT models**.**

The base DiT models took the following form:

$$log(ED\_mny) = \beta_0 + \beta_1 Temp\_mny + \beta_2 Precip\_mny + \beta_3 PM_{2.5}\_mny + \alpha\_m + \gamma\_n + \delta\_y + log(Pop\_mny)$$

Where:

β_0: Model intercept representing the average log emergency department (ED) visit rate, among those ages 18 to 64, in municipality m in month n and year y, when all predictors are zero.

β_1: Slope coefficient representing the association between monthly average of maximum or minimum daily temperatures and average log emergency department (ED) visit rate among those ages 18 to 64 in municipality m in month n and year y,

β_2: Slope coefficient representing the association between monthly average precipitation (Precip_mny) and average log emergency department (ED) visit rate, among those ages 18 to 64, in municipality m in month n and year y

β_3 : Slope coefficient representing the association between monthly $PM_{2.5}$ concentration (PM_{25}_mny) and average log ED visit rate, among those ages 18 to 64, in municipality m in month n and year y

ED_mny : Counts of ED visits among those ages 18-64 in municipality *m* in month *n* and year *y*.

α_m : Municipality fixed effects, capturing time-invariant differences between municipalities.

γ_n : Month fixed effects, controlling for seasonal patterns.

δ_y : Year fixed effects, controlling for broader temporal trends.

log(Pop_ny) : Population offset for each municipality m in year n.

Next, in the DiDiT models, we included an interaction term between a policy enactment indicator, coded 1 for years 2014-2016 (the years after which all of the treated municipalities had enacted the PSL policy) and 0 otherwise (years 2011-2013), and the temperature metric, to estimate the change in the association between heat and ED visit rates following policy enactment. The DiDiT models appeared as follows:

$$log(ED\_mny) = \beta_0 + \beta_1 Temp\_mny + \beta_2 Precip\_mny + \beta_3 PM_{2.5}\_mny + \beta_4 Temp\_mny * PSL + \alpha\_m + \gamma\_n + \delta\_y + log(Pop\_mny)$$

$Temp\_mny * PSL$ is the DiDiT estimator, representing the difference in the slope-- representing the temperature-ED visit rate association -following the enactment of the PSL policies.

Appendix C. Distribution of covariates, at baseline, in treated and comparison municipalities in New Jersey.

Municipality	Population density 2013	Mean max temperature	Mean minimum temperature	Percent living in poverty 2013
**TREATED**				
Jersey City	6569.80	26.08	18.61	0.18
Newark	4426.44	26.76	17.74	0.29
**UNTREATED**				
Hackensack City	4016.00	26.68	17.54	0.17
Woodlynne Borough	5229.99	27.98	18.54	0.29
City of Orange Township	5268.02	26.25	16.43	0.22
Harrison Town	4500.23	26.90	17.77	0.18
Perth Amboy City	4212.43	27.08	17.18	0.24

Appendix D. Event time plot representing the treated vs. comparison municipality difference in the association between monthly averages of minimum daily temperatures and the natural logarithm of ED visits (95% CI) in adults ages 18-64, for each year before the paid sick leave policy was adopted in the treated municipalities.

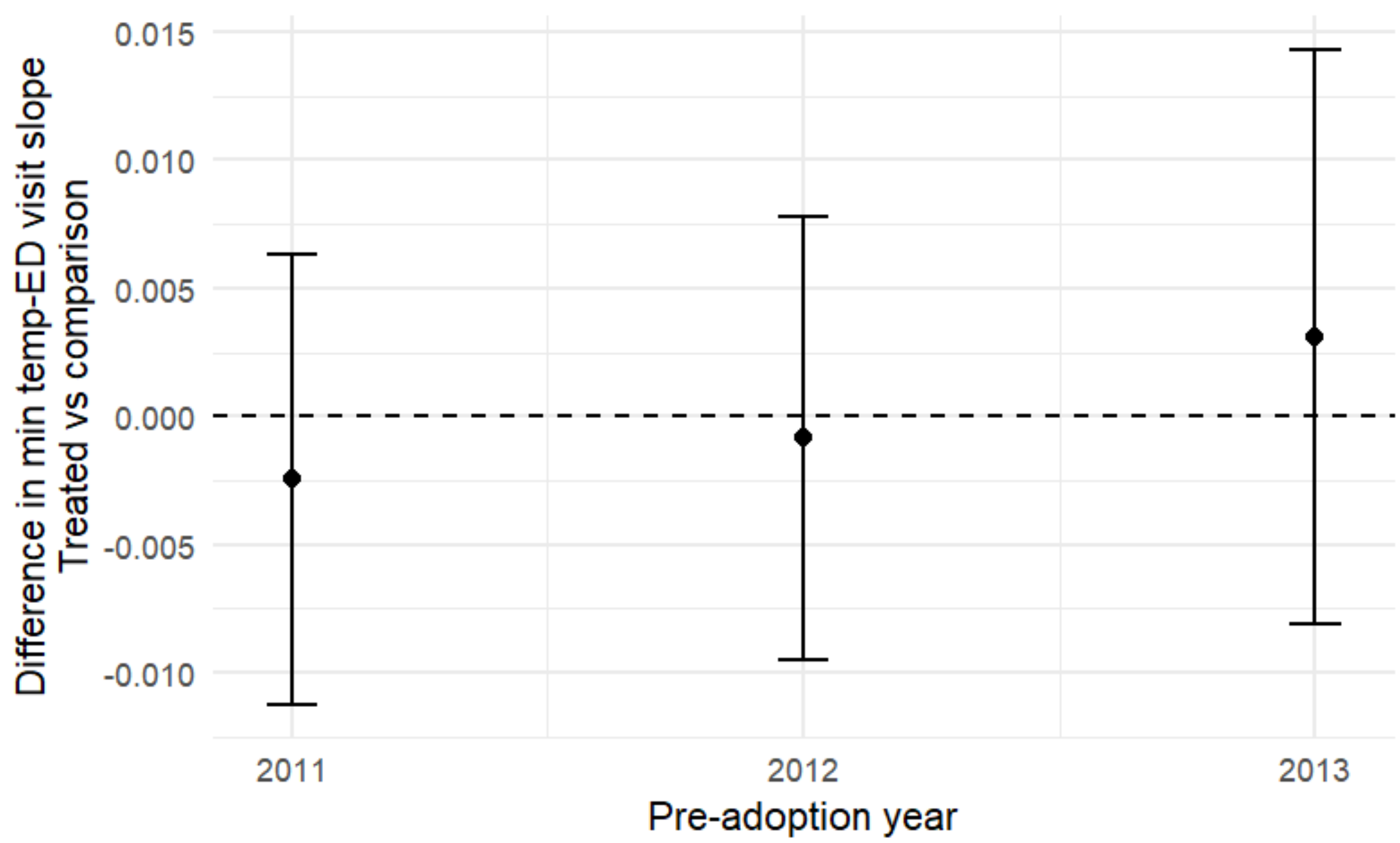

Appendix E. Details on the DiDiDiT models.

The full DiDiDiT model appeared as follows:

$$log(ED\_mny) = \beta_0 + \beta_1 Temp\_mny + \beta_2 Precip\_mny + \beta_3 PM_{2.5}\_mny + \beta_4 Temp\_mny * PSL + \beta_5 PSL * treated + \beta_6 Temp\_mny * treated + \beta_7 Temp\_mny * PSL * treated + \alpha\_m + \gamma\_n + \delta\_y + log(Pop\_mny)$$

Here, the treated term represents if the municipality enacted a PSL policy in year 2014. β_7 is the DiDiDiT estimator, representing the difference in the slope representing the association between the temperature metric and average log ED visit rates, following the enactment of the PSL policies, comparing treated with untreated municipalities.

Appendix F. Total monthly counts of all cause ED visits in those ages 18-64, May-September, 2011-2016, stratified by NJ municipality, year, and month.

Municipality	Year	Month	Total ED visits
City of Orange Township	2011	May	4536
City of Orange Township	2011	June	4690
City of Orange Township	2011	July	4256
City of Orange Township	2011	August	4305
City of Orange Township	2011	September	4011
City of Orange Township	2012	May	4564
City of Orange Township	2012	June	4508
City of Orange Township	2012	July	4634
City of Orange Township	2012	August	4739
City of Orange Township	2012	September	4522
City of Orange Township	2013	May	4928
City of Orange Township	2013	June	4585
City of Orange Township	2013	July	5124
City of Orange Township	2013	August	4760
City of Orange Township	2013	September	4193
City of Orange Township	2014	May	4704
City of Orange Township	2014	June	4788
City of Orange Township	2014	July	4886
City of Orange Township	2014	August	5026
City of Orange Township	2014	September	5789
City of Orange Township	2015	May	7567
City of Orange Township	2015	June	6608
City of Orange Township	2015	July	6587
City of Orange Township	2015	August	7448
City of Orange Township	2015	September	6587
City of Orange Township	2016	May	7112
City of Orange Township	2016	June	6909
City of Orange Township	2016	July	6797
City of Orange Township	2016	August	7280
City of Orange Township	2016	September	6678
Hackensack City	2011	May	7385
Hackensack City	2011	June	7406
Hackensack City	2011	July	7441
Hackensack City	2011	August	7469
Hackensack City	2011	September	6510
Hackensack City	2012	May	7077
Hackensack City	2012	June	7035

Hackensack City	2012	July	7973
Hackensack City	2012	August	7511
Hackensack City	2012	September	6769
Hackensack City	2013	May	6979
Hackensack City	2013	June	7315
Hackensack City	2013	July	7784
Hackensack City	2013	August	6846
Hackensack City	2013	September	7266
Hackensack City	2014	May	6930
Hackensack City	2014	June	6461
Hackensack City	2014	July	7203
Hackensack City	2014	August	7518
Hackensack City	2014	September	6909
Hackensack City	2015	May	7336
Hackensack City	2015	June	7553
Hackensack City	2015	July	7728
Hackensack City	2015	August	7574
Hackensack City	2015	September	7497
Hackensack City	2016	May	7014
Hackensack City	2016	June	7406
Hackensack City	2016	July	7679
Hackensack City	2016	August	7462
Hackensack City	2016	September	6958
Harrison Town	2011	May	1393
Harrison Town	2011	June	1330
Harrison Town	2011	July	1540
Harrison Town	2011	August	1652
Harrison Town	2011	September	1456
Harrison Town	2012	May	1729
Harrison Town	2012	June	1484
Harrison Town	2012	July	1512
Harrison Town	2012	August	1694
Harrison Town	2012	September	1365
Harrison Town	2013	May	1862
Harrison Town	2013	June	1407
Harrison Town	2013	July	1799
Harrison Town	2013	August	1757
Harrison Town	2013	September	1680
Harrison Town	2014	May	1351
Harrison Town	2014	June	1652
Harrison Town	2014	July	1708
Harrison Town	2014	August	1764
Harrison Town	2014	September	1715
Harrison Town	2015	May	1743

Harrison Town	2015	June	1512
Harrison Town	2015	July	1582
Harrison Town	2015	August	1820
Harrison Town	2015	September	1820
Harrison Town	2016	May	1925
Harrison Town	2016	June	1757
Harrison Town	2016	July	1638
Harrison Town	2016	August	1400
Harrison Town	2016	September	1652
Jersey City	2011	May	31003
Jersey City	2011	June	31892
Jersey City	2011	July	33411
Jersey City	2011	August	31192
Jersey City	2011	September	30310
Jersey City	2012	May	33159
Jersey City	2012	June	31780
Jersey City	2012	July	35413
Jersey City	2012	August	34965
Jersey City	2012	September	31864
Jersey City	2013	May	40628
Jersey City	2013	June	40747
Jersey City	2013	July	46788
Jersey City	2013	August	41314
Jersey City	2013	September	37744
Jersey City	2014	May	41594
Jersey City	2014	June	42931
Jersey City	2014	July	44044
Jersey City	2014	August	45682
Jersey City	2014	September	42574
Jersey City	2015	May	45927
Jersey City	2015	June	45304
Jersey City	2015	July	46403
Jersey City	2015	August	48944
Jersey City	2015	September	46431
Jersey City	2016	May	43617
Jersey City	2016	June	43232
Jersey City	2016	July	46431
Jersey City	2016	August	45409
Jersey City	2016	September	43057
Newark City	2011	May	69160
Newark City	2011	June	68432
Newark City	2011	July	69237
Newark City	2011	August	67690
Newark City	2011	September	63868

Newark City	2012	May	72219
Newark City	2012	June	68642
Newark City	2012	July	72989
Newark City	2012	August	73878
Newark City	2012	September	67102
Newark City	2013	May	67095
Newark City	2013	June	68579
Newark City	2013	July	73262
Newark City	2013	August	70721
Newark City	2013	September	67970
Newark City	2014	May	69811
Newark City	2014	June	69468
Newark City	2014	July	72303
Newark City	2014	August	72296
Newark City	2014	September	74529
Newark City	2015	May	78778
Newark City	2015	June	78204
Newark City	2015	July	81697
Newark City	2015	August	81410
Newark City	2015	September	80668
Newark City	2016	May	79877
Newark City	2016	June	78386
Newark City	2016	July	79604
Newark City	2016	August	83748
Newark City	2016	September	74823
Perth Amboy City	2011	May	11249
Perth Amboy City	2011	June	11697
Perth Amboy City	2011	July	11774
Perth Amboy City	2011	August	11641
Perth Amboy City	2011	September	10927
Perth Amboy City	2012	May	11739
Perth Amboy City	2012	June	11704
Perth Amboy City	2012	July	12628
Perth Amboy City	2012	August	12411
Perth Amboy City	2012	September	11802
Perth Amboy City	2013	May	12110
Perth Amboy City	2013	June	11739
Perth Amboy City	2013	July	13636
Perth Amboy City	2013	August	12537
Perth Amboy City	2013	September	11711
Perth Amboy City	2014	May	12320
Perth Amboy City	2014	June	11991
Perth Amboy City	2014	July	12831
Perth Amboy City	2014	August	13650

Perth Amboy City	2014	September	12474
Perth Amboy City	2015	May	13139
Perth Amboy City	2015	June	12845
Perth Amboy City	2015	July	13223
Perth Amboy City	2015	August	13307
Perth Amboy City	2015	September	12642
Perth Amboy City	2016	May	13090
Perth Amboy City	2016	June	12943
Perth Amboy City	2016	July	13286
Perth Amboy City	2016	August	13916
Perth Amboy City	2016	September	14021
Woodlynne Borough	2011	May	658
Woodlynne Borough	2011	June	602
Woodlynne Borough	2011	July	560
Woodlynne Borough	2011	August	777
Woodlynne Borough	2011	September	686
Woodlynne Borough	2012	May	651
Woodlynne Borough	2012	June	707
Woodlynne Borough	2012	July	700
Woodlynne Borough	2012	August	777
Woodlynne Borough	2012	September	546
Woodlynne Borough	2013	May	602
Woodlynne Borough	2013	June	602
Woodlynne Borough	2013	July	581
Woodlynne Borough	2013	August	623
Woodlynne Borough	2013	September	532
Woodlynne Borough	2014	May	539
Woodlynne Borough	2014	June	630
Woodlynne Borough	2014	July	511
Woodlynne Borough	2014	August	602
Woodlynne Borough	2014	September	595
Woodlynne Borough	2015	May	560
Woodlynne Borough	2015	June	623
Woodlynne Borough	2015	July	700
Woodlynne Borough	2015	August	637
Woodlynne Borough	2015	September	742
Woodlynne Borough	2016	May	609
Woodlynne Borough	2016	June	595
Woodlynne Borough	2016	July	693
Woodlynne Borough	2016	August	728
Woodlynne Borough	2016	September	525

Appendix G. Rate ratio (RR) and 95% CI estimates of association between monthly averages of minimum and maximum temperatures and rates of ED visits. Results shown are from sensitivity analyses in which: (A) policy adoption dates were altered to 2013 and 2015; (B) DiDiT analyses were run separately for the two treated municipalities, Jersey City and Newark City; (C) the PSL policy adoption date was changed from May 2014 to July 2014; and (D) DiDiT analyses were conducted using monthly counts all-age ED visits, rather than those that occurred among those ages 18-64, as the dependent variable.

Analysis	Sample	Temperature	Estimator	RR	LL	UL
A: Placebo policy adoption in 2013	2 treated municipalities	Minimum temperature	Pre-enactment DiT	0.995	0.965	1.026
			Post-enactment DiT	1.006	0.989	1.024
			Difference in post versus pre-enactment DiT	1.011	0.992	1.031
		Maximum temperature	Pre-enactment DiT	1.009	0.990	1.028
			Post-enactment DiT	1.006	0.994	1.019
			Difference in post versus pre-enactment DiT	0.998	0.982	1.014
A: Placebo policy adoption in 2015		Minimum temperature	Pre-enactment DiT	1.015	0.994	1.036
			Post-enactment DiT	1.012	0.995	1.030
			Difference in post versus pre-enactment DiT	0.997	0.986	1.008
		Maximum temperature	Pre-enactment DiT	1.011	0.995	1.028
			Post-enactment DiT	1.006	0.993	1.019
			Difference in post versus pre-enactment DiT	0.995	0.984	1.006
B: Models run separately for each treated municipality	Jersey City	Minimum temperature	Pre-enactment DiT	1.022	1.005	1.040
			Post-enactment DiT	1.019	1.003	1.035
			Difference in post versus pre-enactment DiT	0.997	0.992	1.002
		Maximum temperature	Pre-enactment DiT	1.015	0.997	1.034
			Post-enactment DiT	1.013	1.000	1.027
			Difference in post versus pre-enactment DiT	0.998	0.990	1.005
	Newark City	Minimum temperature	Pre-enactment DiT	1.010	1.001	1.020
			Post-enactment DiT	1.008	0.999	1.017
			Difference in post versus pre-enactment DiT	0.998	0.992	1.003

Analysis	Sample	Temperature	Estimator	RR	LL	UL
		Maximum temperature	Pre-enactment DiT	1.007	0.994	1.020
			Post-enactment DiT	1.005	0.995	1.014
			Difference in post versus pre-enactment DiT	0.998	0.990	1.005
C: July 2014 rather than May 2014 policy adoption	2 treated municipalities	Minimum temperature	Pre-enactment DiT	1.016	0.996	1.037
			Post-enactment DiT	1.014	0.995	1.033
			Difference in post versus pre-enactment DiT	0.998	0.987	1.009
		Maximum temperature	Pre-enactment DiT	1.014	0.997	1.031
			Post-enactment DiT	1.006	0.992	1.020
			Difference in post versus pre-enactment DiT	0.992	0.982	1.003
D: Models run using counts of all-age ED visits as the outcome		Minimum temperature	Pre-enactment DiT	1.011	0.990	1.032
			Post-enactment DiT	1.010	0.992	1.028
			Difference in post versus pre-enactment DiT	0.999	0.988	1.011
		Maximum temperature	Pre-enactment DiT	1.014	0.999	1.030
			Post-enactment DiT	1.006	0.995	1.018
			Difference in post versus pre-enactment DiT	0.992	0.980	1.004

Appendix H. DiDiDiT analysis results representing the relative difference in the post versus pre-enactment association between monthly averages of minimum and maximum daily temperatures, in treated versus comparison municipalities in NJ. In this sensitivity analysis, comparison 3, 8, and 12 municipalities were selected using ±5%, 20%, and 30% thresholds, respectively, for baseline characteristics, rather than the primary ±10% threshold.

Threshold	Sample	Temperature	Estimator	RR	LL	UL
5%	2 treated plus 3 comparison municipalities	Minimum temperature	Pre-enactment DiT, Comparison	1.015	0.997	1.033
			Pre-enactment DiT, Treated	1.011	0.994	1.028
			Post-enactment DiT, Comparison	1.007	0.992	1.023
			Post-enactment DiT, Treated	1.011	0.997	1.026
			Difference in post versus pre-enactment slopes, Comparison	0.993	0.983	1.002
			Difference in post versus pre-enactment slopes, Treated	1.000	0.989	1.012
			DiDiDiT estimate	1.008	0.996	1.019
		Maximum temperature	Pre-enactment DiT, Comparison	1.013	0.998	1.028
			Pre-enactment DiT, Treated	1.011	0.997	1.026
			Post-enactment DiT, Comparison	1.002	0.990	1.014
			Post-enactment DiT, Treated	1.004	0.995	1.015
			Difference in post versus pre-enactment slopes, Comparison	0.989	0.978	1.000
			Difference in post versus pre-enactment slopes, Treated	0.993	0.982	1.005
			DiDiDiT estimate	1.004	0.993	1.015
20%	2 treated plus 8 comparison municipalities	Minimum temperature	Pre-enactment DiT, Comparison	1.016	1.001	1.031
			Pre-enactment DiT, Treated	1.011	0.996	1.026
			Post-enactment DiT, Comparison	1.005	0.991	1.020
			Post-enactment DiT, Treated	1.010	0.998	1.022
			Difference in post versus pre-enactment slopes, Comparison	0.989	0.979	0.999
			Difference in post versus pre-enactment slopes, Treated	0.999	0.988	1.010
			DiDiDiT estimate	1.010	0.997	1.022
		Maximum temperature	Pre-enactment DiT, Comparison	1.016	1.003	1.030
			Pre-enactment DiT, Treated	1.015	1.002	1.029

Threshold	Sample	Temperature	Estimator	RR	LL	UL
			Post-enactment DiT, Comparison	1.010	0.997	1.023
			Post-enactment DiT, Treated	1.007	0.998	1.017
			Difference in post versus pre-enactment slopes, Comparison	0.993	0.983	1.004
			Difference in post versus pre-enactment slopes, Treated	0.992	0.982	1.003
			DiDiDiT estimate	0.999	0.987	1.011
30%	2 treated plus 12 comparison municipalities	Minimum temperature	Pre-enactment DiT, Comparison	1.013	1.000	1.026
			Pre-enactment DiT, Treated	1.010	0.997	1.024
			Post-enactment DiT, Comparison	1.006	0.994	1.018
			Post-enactment DiT, Treated	1.009	0.999	1.020
			Difference in post versus pre-enactment slopes, Comparison	0.992	0.985	1.000
			Difference in post versus pre-enactment slopes, Treated	0.999	0.989	1.009
			DiDiDiT estimate	1.007	0.996	1.018
		Maximum temperature	Pre-enactment DiT, Comparison	1.015	1.003	1.026
			Pre-enactment DiT, Treated	1.015	1.003	1.027
			Post-enactment DiT, Comparison	1.008	0.998	1.018
			Post-enactment DiT, Treated	1.007	0.999	1.016
			Difference in post versus pre-enactment slopes, Comparison	0.994	0.985	1.002
			Difference in post versus pre-enactment slopes, Treated	0.993	0.983	1.003
			DiDiDiT estimate	0.999	0.989	1.010

RR = rate ratio; LL = lower limit; UL = upper limit. All 95% confidence intervals use calendar-gap-aware Newey-West inference, the fixest 0.12.1 small-sample correction, and the model-specific Student's t critical value.

Appendix I. DiDiDiT analysis results representing the relative difference in the post versus pre-enactment association between monthly averages of minimum and maximum daily temperatures, in treated versus comparison municipalities in NJ. In these sensitivity analyses, Newey-West (NW) bandwidths of 1, 3, 4, 8 and 12 calendar months, instead of 2, were specified.

NW lag	Temperature	Estimator	RR	LL	UL
1	Minimum temperature	Pre-enactment DiT, Comparison	1.016	0.998	1.034
		Pre-enactment DiT, Treated	1.010	0.994	1.028
		Post-enactment DiT, Comparison	1.005	0.988	1.022
		Post-enactment DiT, Treated	1.010	0.997	1.024
		Difference in post versus pre-enactment slopes, Comparison	0.989	0.977	1.002
		Difference in post versus pre-enactment slopes, Treated	1.000	0.988	1.012
		DiDiDiT estimate	1.011	0.996	1.026
	Maximum temperature	Pre-enactment DiT, Comparison	1.015	0.998	1.031
		Pre-enactment DiT, Treated	1.012	0.997	1.029
		Post-enactment DiT, Comparison	1.004	0.990	1.020
		Post-enactment DiT, Treated	1.005	0.995	1.016
		Difference in post versus pre-enactment slopes, Comparison	0.990	0.977	1.004
		Difference in post versus pre-enactment slopes, Treated	0.993	0.981	1.005
		DiDiDiT estimate	1.003	0.988	1.018
2	Minimum temperature	Pre-enactment DiT, Comparison	1.016	1.000	1.032
		Pre-enactment DiT, Treated	1.010	0.995	1.026
		Post-enactment DiT, Comparison	1.005	0.989	1.021
		Post-enactment DiT, Treated	1.010	0.998	1.023
		Difference in post versus pre-enactment slopes, Comparison	0.989	0.978	1.000
		Difference in post versus pre-enactment slopes, Treated	1.000	0.989	1.011
		DiDiDiT estimate	1.011	0.998	1.024
	Maximum temperature	Pre-enactment DiT, Comparison	1.015	1.001	1.029
		Pre-enactment DiT, Treated	1.012	0.999	1.026
		Post-enactment DiT, Comparison	1.004	0.991	1.018
		Post-enactment DiT, Treated	1.005	0.996	1.015
		Difference in post versus pre-enactment slopes, Comparison	0.990	0.978	1.002

NW lag	Temperature	Estimator	RR	LL	UL
		Difference in post versus pre-enactment slopes, Treated	0.993	0.982	1.004
		DiDiDiT estimate	1.003	0.990	1.016
3	Minimum temperature	Pre-enactment DiT, Comparison	1.016	1.002	1.030
		Pre-enactment DiT, Treated	1.010	0.996	1.025
		Post-enactment DiT, Comparison	1.005	0.990	1.019
		Post-enactment DiT, Treated	1.010	0.998	1.022
		Difference in post versus pre-enactment slopes, Comparison	0.989	0.979	0.999
		Difference in post versus pre-enactment slopes, Treated	1.000	0.990	1.010
		DiDiDiT estimate	1.011	0.999	1.023
	Maximum temperature	Pre-enactment DiT, Comparison	1.015	1.002	1.028
		Pre-enactment DiT, Treated	1.012	1.000	1.025
		Post-enactment DiT, Comparison	1.004	0.992	1.017
		Post-enactment DiT, Treated	1.005	0.996	1.014
		Difference in post versus pre-enactment slopes, Comparison	0.990	0.979	1.001
		Difference in post versus pre-enactment slopes, Treated	0.993	0.983	1.003
		DiDiDiT estimate	1.003	0.991	1.014
4	Minimum temperature	Pre-enactment DiT, Comparison	1.016	1.002	1.030
		Pre-enactment DiT, Treated	1.010	0.997	1.024
		Post-enactment DiT, Comparison	1.005	0.991	1.018
		Post-enactment DiT, Treated	1.010	0.999	1.022
		Difference in post versus pre-enactment slopes, Comparison	0.989	0.979	0.999
		Difference in post versus pre-enactment slopes, Treated	1.000	0.990	1.009
		DiDiDiT estimate	1.011	1.000	1.022
	Maximum temperature	Pre-enactment DiT, Comparison	1.015	1.002	1.027
		Pre-enactment DiT, Treated	1.012	1.001	1.024
		Post-enactment DiT, Comparison	1.004	0.993	1.016
		Post-enactment DiT, Treated	1.005	0.997	1.014
		Difference in post versus pre-enactment slopes, Comparison	0.990	0.979	1.001
		Difference in post versus pre-enactment slopes, Treated	0.993	0.984	1.002
		DiDiDiT estimate	1.003	0.992	1.014

NW lag	Temperature	Estimator	RR	LL	UL
8	Minimum temperature	Pre-enactment DiT, Comparison	1.016	1.004	1.028
		Pre-enactment DiT, Treated	1.010	0.998	1.023
		Post-enactment DiT, Comparison	1.005	0.993	1.017
		Post-enactment DiT, Treated	1.010	1.000	1.021
		Difference in post versus pre-enactment slopes, Comparison	0.989	0.980	0.998
		Difference in post versus pre-enactment slopes, Treated	1.000	0.991	1.008
		DiDiDiT estimate	1.011	1.001	1.021
	Maximum temperature	Pre-enactment DiT, Comparison	1.015	1.004	1.026
		Pre-enactment DiT, Treated	1.012	1.002	1.023
		Post-enactment DiT, Comparison	1.004	0.995	1.014
		Post-enactment DiT, Treated	1.005	0.998	1.013
		Difference in post versus pre-enactment slopes, Comparison	0.990	0.981	1.000
		Difference in post versus pre-enactment slopes, Treated	0.993	0.985	1.000
		DiDiDiT estimate	1.003	0.994	1.012
12	Minimum temperature	Pre-enactment DiT, Comparison	1.016	1.005	1.027
		Pre-enactment DiT, Treated	1.010	0.999	1.022
		Post-enactment DiT, Comparison	1.005	0.994	1.016
		Post-enactment DiT, Treated	1.010	1.001	1.020
		Difference in post versus pre-enactment slopes, Comparison	0.989	0.981	0.997
		Difference in post versus pre-enactment slopes, Treated	1.000	0.992	1.007
		DiDiDiT estimate	1.011	1.002	1.020
	Maximum temperature	Pre-enactment DiT, Comparison	1.015	1.004	1.025
		Pre-enactment DiT, Treated	1.012	1.003	1.022
		Post-enactment DiT, Comparison	1.004	0.996	1.013
		Post-enactment DiT, Treated	1.005	0.998	1.013
		Difference in post versus pre-enactment slopes, Comparison	0.990	0.982	0.999
		Difference in post versus pre-enactment slopes, Treated	0.993	0.986	1.000
		DiDiDiT estimate	1.003	0.995	1.011

RR = rate ratio; LL = lower limit; UL = upper limit. All 95% confidence intervals use calendar-gap-aware Newey-West inference, the fixest 0.12.1 small-sample correction, and the model-specific Student's t critical value.
